# Context Prediction Analysis and Episodic Memory

**DOI:** 10.3389/fnbeh.2013.00132

**Published:** 2013-10-07

**Authors:** Sheri J. Y. Mizumori

**Affiliations:** ^1^Laboratory of Neural Systems, Decision Science, Learning and Memory, Department of Psychology, University of Washington, Seattle, WA, USA

**Keywords:** hippocampus, prediction errors, prefrontal cortex, dopamine, striatum, memory, decision making

## Abstract

Events that happen at a particular place and time come to define our episodic memories. Extensive experimental and clinical research illustrate that the hippocampus is central to the processing of episodic memories, and this is in large part due to its analysis of context information according to spatial and temporal references. In this way, hippocampus defines ones expectations for a given context as well as detects errors in predicted contextual features. The detection of context prediction errors is hypothesized to distinguished events into meaningful epochs that come to be recalled as separate episodic memories. The nature of the spatial and temporal context information processed by hippocampus is described, as is a hypothesis that the apparently self-regulatory nature of hippocampal context processing may ultimately be mediated by natural homeostatic operations and plasticity. Context prediction errors by hippocampus are suggested to be valued by the midbrain dopamine system, the output of which is ultimately fed back to hippocampus to update memory-driven context expectations for future events. Thus, multiple network functions (both within and outside hippocampus) combine to result in adaptive episodic memories.

## Introduction

Events are typically defined by the situations that are associated with significant outcomes. Each situation, or context, is multifaceted in that it includes not only the external sensory environment, but also our intrinsic motivational and emotional state, as well as social considerations. Thus it should not be surprising that the cells of the hippocampus, long thought to mediate episodic or event-based memories (e.g., O’Keefe and Nadel, 1978; Tulving, 2002), have been found to represent a variety of context-defining information. The challenge has been, however, to understand how these hippocampal representations of context-specific information are used to generate episodic memories. The following discusses the view that hippocampus represents context information in order to determine whether the expected contextual features match those currently being experienced. The identification of mismatches (termed *context prediction errors*) may lead to a cascading series of assessments through connected brain regions that could ultimately alter future decisions and update memories.

## Spatial Context-Dependency of Hippocampal Neural Codes

It is generally accepted that hippocampus processes contextual information (e.g., Hirsh, 1974; Myers and Gluck, 1994; Anagnostaras et al., 2001; Maren, 2001; Fanselow and Poulos, 2005; Bouton et al., 2006). There are numerous demonstrations in which conditioned responses to contextual stimuli are eliminated with hippocampal damage while responses to discrete conditioned stimuli are unaffected (e.g., Kim and Fanselow, 1992; Phillips and LeDoux, 1992, 1994). Also, animals with hippocampal or entorhinal cortical damage do not show the normal decrement in conditioned responding after a shift in context (Penick and Solomon, 1991; Freeman et al., 1996a,b). The hippocampus of freely behaving animals is predisposed to represent context information within a spatial framework: during unrestrained navigation, hippocampal neurons fire selectively as animals traverse restricted areas of their environment, referred to as *place fields* (O’Keefe and Dostrovsky, 1971). As decades of research have shown (summarized in Mizumori, 2008), place fields are dynamic and integrated representations of multiple types of context-defining information. For example, changing any modality of cues, the motivational state, or the behaviors needed to perform the task result in alterations of place field properties, a process commonly referred to as *remapping*.

The readiness of place fields to remap is evident when changes are made to the visual environment (e.g., Ranck, 1973; O’Keefe, 1976; Olton et al., 1978; Muller and Kubie, 1987), such as its geometric features (e.g., Gothard et al., 1996; O’Keefe and Burgess, 1996; Wiener, 1996). Other sensory inputs also bias place field activity, including olfactory (Save et al., 2000), auditory (O’Keefe and Conway, 1978; McEchron and Disterhoft, 1999), and somatosensory information (Young et al., 1994). Thus, an extensive literature (Mizumori, 2008) verifies that hippocampal pyramidal neurons process multimodal sensory input. Hippocampal place fields are also sensitive to changes in a task’s reward structure (Smith and Mizumori, 2006b; Wikenheiser and Redish, 2011). Smith and Mizumori (2006b) explicitly tested this idea by training rats to distinguish Context A from Context B according to where reward was expected to be found. The motivational, sensory, and behavioral requirements of task performance were purposely held constant across the two contexts so that changes in place fields could be attributed to the recall of a different memory. Place fields remapped at the beginning of trials in Context B, a time when there is heightened uncertainty about the context conditions. In a similar experiment, Wikenheiser and Redish (2011) demonstrated that changes in reward contingency can modulate the trial-to-trial variability of hippocampal place cell activity, again suggesting that uncertainty can drive place field remapping. Finally, the relative contributions of sensory, motivation (including reward), or response information to a given place field vary with task demands, and this is evidenced by findings across many laboratories that place fields change when rats use identical information to solve tasks according to different mnemonic strategies (e.g., Ferbinteanu and Shapiro, 2003; Mizumori et al., 2004; Eschenko and Mizumori, 2007).

In sum, it is clear that place fields can come to represent different types of sensory, behavioral, and intrinsic information that have strong spatial and contextual (i.e., experience-dependent) features, thereby validating the term *spatial context* when referring to the informational content of place fields (Nadel et al., 1985; Mizumori et al., 1999, 2000; Jeffery et al., 2004). If this information indeed underlies the effective use and generation of episodic memories, Nadel (2008) has argued that the neural representations that define a familiar context must be relatively stable and predictable, maintaining their relationship to each other, until prediction errors are detected. This pattern of place field responses has indeed been documented in the literature. Stable place fields, then, can be said to reflect *the integration of stable background sensory information, internal state or motivational information, as well as response or behavioral outcome expectancies within a spatial framework as a function of time.*

To the extent that different cognitive strategies are mediated by different underlying memories, one network pattern of activated place cells is thought to reflect one memory, and a different pattern of activated place cells corresponds to a different memory (e.g., Samsonovich and McNaughton, 1997). When one refers to place field remapping, then, implicit is the notion that each map (or collection of activated place cells) is driven by a different memory.

## Spatial Context Discrimination and Prediction

As striking as location-selective firing is to even the naive observer, equally impressive is the fact that the otherwise stable place fields readily remap when most any feature of the spatial context changes. This sensitivity to changes in context has led many to suggest that a fundamental operation of the hippocampus is to detect changes in contextual information so that animals can discriminate contexts (e.g., Smith and Mizumori, 2006a,b). The ability to distinguish contexts likely relies on computations that are often attributed to hippocampus, including those that underlie the flexible use of conjunctive, sequential, relational, and spatial algorithms (e.g., O’Keefe and Nadel, 1978; Foster et al., 1987; Eichenbaum et al., 1999; Wood et al., 2000; Eichenbaum and Cohen, 2001; O’Reilly and Rudy, 2001; Fortin et al., 2002). The latter, in turn, are thought to be supported by various forms of pattern separation and pattern completion neurocomputations (Mizumori et al., 2004, 2007b; Penner and Mizumori, 2012a,b).

A *Context Discrimination Hypothesis* (CDH) postulates that single hippocampal neuronal representations of context provide multidimensional data to population-based network computations that ultimately determine whether expected contextual features of a situation have changed (e.g., Mizumori et al., 1999, 2000, 2007a; Smith and Mizumori, 2006a,b; Mizumori, 2008). Initial suggestive evidence of this interpretation of hippocampal network function was the repeated observation that upon less than complete changes in a familiar context, many but not all place fields remap (e.g., Tanila et al., 1997; Mizumori et al., 1999; Brown and Skaggs, 2002; Knierim, 2002; Lee et al., 2004). The place fields that remained in the face of changes in a familiar context were considered to represent the stable or expected contextual features. The place fields that changed, then, could be thought of as representing current context information. The existence of these two types of place field responses gave rise to the notion that hippocampus compares expected and experienced context features (Mizumori et al., 1999). This idea begs the question, then, why does hippocampus represent both expected (learned) and current context information? These hippocampal spatial context representations (O’Keefe and Nadel, 1978; Nadel and Wilner, 1980; Nadel and Payne, 2002) may contribute to a match-mismatch type of analysis that evaluates the present context according to how similar it is to the context that an animal expects to encounter based on past experiences (e.g., Gray, 1982; Vinogradova, 1995; Mizumori et al., 1999, 2000; Gray, 2000; Lisman and Otmakhova, 2001; Hasselmo et al., 2002; Anderson and Jeffery, 2003; Jeffery et al., 2004; Hasselmo, 2005b; Smith and Mizumori, 2006a,b; Manns et al., 2007a; Nadel, 2008). Detected mismatches may be signaled by a change in the pattern of input from hippocampus or possibly by a specific input pattern. This question remains to be answered. Nevertheless, mismatch signals can be used to identify novel situations and to distinguish different contexts, functions that are necessary to define significant events or episodes. Mismatch signals also may engage neural mechanisms that determine the value of the mismatch so that existing memories can be updated and/or new memories can be formed. When context match signals are generated, the effect could be to strengthen currently active memory networks located elsewhere in the brain (e.g., neocortex). In this way, hippocampus may play different mnemonic roles depending on whether or not contexts actually change.

In support of the CDH, disconnecting hippocampus by fornix lesions impairs context discrimination (Smith et al., 2004), and hippocampal lesions reduce animals’ ability to respond to changes in a familiar environment (Good and Honey, 1991; Save et al., 1992a,b). Spatial novelty detection corresponds to selective elevation of the immediate early gene c-*fos* in hippocampus, and not in surrounding parahippocampal cortical regions (Jenkins et al., 2004). Also, as described above, hippocampal neurons show significantly altered firing patterns when rats experience spatial or non-spatial changes in a familiar environment (O’Keefe, 1976; Muller and Kubie, 1987; Wood et al., 1999; Fyhn et al., 2002; Ferbinteanu and Shapiro, 2003; Moita et al., 2004; Yeshenko et al., 2004; Leutgeb et al., 2005a,b; Puryear et al., 2006; Smith and Mizumori, 2006b; Eschenko and Mizumori, 2007). As an example, Smith and Mizumori (2006b) showed that hippocampal neurons develop context-specific responses only when rats were required to discriminate contexts. Discriminating neural responses were not observed when rats were allowed to randomly forage for the same amount of time. Further, Manns et al. (2007b) demonstrated that relative to match trials in an odor cue or object recognition task, CA1 neurons preferentially discharged when animals experienced a non-match situation in these same tasks. Also consistent with the CDH, neuroimaging studies of human performance shows that hippocampus becomes differentially active during match and mismatch trials (Kumaran and Maguire, 2007; Kuhl et al., 2010; Chen et al., 2011; Dickerson et al., 2011; Foerde and Shohamy, 2011; Duncan et al., 2012a,b).

The detection of changes in context is fundamentally important for the continual selection of appropriate behaviors that optimize performance and learning in a variety of tasks (e.g., navigation-based learning, instrumental conditioning, or classical conditioning). Context discrimination engages and prepares cellular mechanisms for rapid and new learning at potentially important times (Paulsen and Moser, 1998), as it is generally known that novelty detection increases attention and exploratory behaviors in a variety of tasks. Interestingly, hippocampal cell firing tends to occur during the “encoding phase” of the ongoing theta rhythm (Hasselmo, 2005a), which is increased during exploratory and investigatory behaviors (Vanderwolf, 1969). Thus, detection of a non-match situation can change the relationship between cell discharge and the local theta rhythm such that encoding functions are enhanced. Detection of matches, on the other hand, does not cause changes in the hippocampal neural activity profile, resulting in efferent messages that continue to retrieve/utilize the currently active memory network that recently drove the execution of successful responses. Context discrimination, then, can be viewed as being critical for the formation of new episodic memories because it leads to the separation in time and space one meaningful event from the next. Such division of memories could facilitate long-term information storage according to memory schemas (Tse et al., 2007; Bethus et al., 2010).

Since hippocampus seems particularly sensitive to changes in the expected (i.e., experience-dependent) context-defining features of a situation, its mismatch signals can be considered to reflect errors in predicted encounters with contextual features, or *context prediction errors*. Indeed, it has been shown that the greater the change in familiar context information, the more place fields remap (e.g., Leutgeb et al., 2005a). It should be noted, however, that when hippocampal cells respond differently to two contexts, it may be because they receive different afferent signals when animals experience different contexts. A second possibility is that the hippocampus receives input from memories stores that define context expectations, and this is actively compared within hippocampus to different input that defines the current context. The simultaneous presence of both familiar and new context information in hippocampus supports the latter possibility. In either case, however, transmission of a context prediction error signal from hippocampus may inform distal brain areas that a change in the context has occurred. Upon receipt of the context prediction error message, efferent midbrain-striatal structures may respond with changes in excitation or inhibition that reflect preparations for, or actual evaluation of, the subjective value of the context prediction error signal (e.g., Mizumori et al., 2004; Lisman and Grace, 2005; Humphries and Prescott, 2010; Penner and Mizumori, 2012a). On the other hand, a hippocampal signal indicating that there was no prediction error may enable plasticity mechanisms that ultimately allow new information to be incorporated into existing memory schemas (e.g., Mizumori et al., 2007a,b; Tse et al., 2007; Bethus et al., 2010). Thus, hippocampal context analyses become critical for the formation of new episodic memories not only because prediction signals provide a mechanism that separates in time and space one meaningful event from the next, but also because the outcome of the prediction error computation engages appropriate neuroplasticity mechanisms in efferent structures that promote subsequent adaptive decisions and memory.

The midbrain dopaminergic system is part of a neural network that assesses the value of behavioral outcomes. Reward-induced excitation of dopamine neurons scales to the magnitude of expected and encountered rewards regardless of the task demands (Schultz et al., 1997; Puryear et al., 2010; Jo et al., 2013): encounters with large rewards are accompanied by larger amplitude phasic dopamine responses than encounters with small amounts of reward. In addition, dopamine cells respond to unexpected reward absences by decreasing their firing rates (Schultz et al., 1997; Puryear et al., 2010). The reduction in firing when rewards are unexpectedly absent is greater if the expectation was for a large, and not small, reward. Further, these reward responses are context-dependent in a manner similar to what is observed for hippocampal place fields (Puryear et al., 2010), a result consistent with the view that hippocampal information guides reward value assessment systems of the brain (e.g., Mizumori et al., 2004; Lisman and Grace, 2005; Puryear et al., 2010) such that the significance of context prediction error messages can be determined (e.g., Penner and Mizumori, 2012a,b). The outcome of the value determination may ultimately come to bias future behavioral responding so that desired goals are more likely to be achieved (see review in Penner and Mizumori, 2012b).

## Context-Dependent Temporal Information Processing and Episodic Memory

Different episodic memories contain information within unique spatial and temporal domains (Tulving, 2002). While there is growing clarity regarding how and why hippocampal neurons represent spatial context information (e.g., Knierim et al., 2006; McNaughton et al., 2006; Penner and Mizumori, 2012a,b; Pilly and Grossberg, 2012; Buzsaki and Moser, 2013), the mechanisms by which spatial context-defined events are distinguished temporally in the service of episodic memory functions remains unclear. However, current evidence support the hypothesis that hippocampus contains an organization structure that permits grouping of information into a number of different time scales (e.g., as reviewed in Buzsaki, 2006; Lisman and Redish, 2009; Grossberg and Pilly, 2013), that are relevant for comparing expected and experienced context information, and thus for episodic memory.

The activity of single neurons and neural networks naturally oscillate within hippocampus and across the brain according to a range of frequencies from low (e.g., 2–4 Hz) to high (e.g., 80 Hz) (Buzsaki, 2006). Oscillatory activity reflects alternating periods of synchronous neural firing: synchronous activity is associated with greater synaptic plasticity and stronger coupling amongst cells of an ensemble, while desynchronous periods are associated with less plasticity and weak signal strength (Hasselmo et al., 2002; Hasselmo, 2005a; Buzsaki, 2006). Thus, inherent in oscillatory neural activity is the ability to segregate on a (very short) time scale the processing of event-related information. In fact, the precise temporal alignment of place cell firing relative to the phase of the ongoing (theta) rhythm processes as animals move through the cell’s place field (referred to as *phase precession*; O’Keefe and Recce, 1993) suggests the possibility that *context-dependent changes in place cell activity alters the state of synaptic plasticity in hippocampus according to experience*. By extension, then, an important impact of altered contextual features is the corresponding change in the nature and/or efficiency of information read into and passed on from hippocampus. Importantly, hippocampal oscillations may enable more temporally precise context representations, and therefore episodic memory. This in turn may enable more precise detection of unexpected context information.

Hippocampal circuits are also observed to synchronize their activity at frequencies greater than the theta frequency. Of particular interest is the gamma band (30–80 Hz) which has been observed in many sensory and motor areas of cortex, hippocampus, parietal cortex, and striatum (e.g., Leung and Yim, 1993; Brosch et al., 2002; Csicsvari et al., 2003; Berke et al., 2004; Bauer et al., 2006; Hoogenboom et al., 2006; Womelsdorf et al., 2006). Orchestration of both excitatory and inhibitory networks within each structure underlies the generation of synchronized gamma oscillations (e.g., Whittington et al., 1995; Vida et al., 2006). Although the functional importance of gamma oscillations remains debated, information carried by the cells that participate in a gamma-burst is effectively accentuated against a background of disorganized neural activity. Therefore, it has been suggested that gamma-bursts represent a fundamental mechanism by which information becomes segmented, filtered, or highlighted within a structure, as well as a mechanisms by which to coordinate information across structures (Buzsaki, 2006). Theta and gamma have many common physiological and behavioral relationships, suggesting that they are components of a coordinated and larger scale oscillatory network. For example, similar to theta rhythms, single unit responses that are recorded simultaneously with gamma oscillations have been found to have specific phase relationships to the gamma rhythm, and both theta and gamma are at least in part regulated by dopamine (e.g., Berke, 2009; van der Meer and Redish, 2009; Kalenscher et al., 2010). Therefore, changes in context that induce remapping (i.e., that altered the constellation of activated neurons), likely change patterns of gamma activity that are characteristic of a familiar context. Since gamma oscillations may effectively select salient information that ultimately impacts decisions, learning, and behavioral responses (e.g., van der Meer and Redish, 2009; Kalenscher et al., 2010), it is predicted that changes in gamma oscillatory patterns are likely to alter future decisions and learning. Further, gamma-bursts should become more predictable as learning takes place within a given context. *The relationship between place fields and the overriding theta and gamma rhythms is then an important mechanism by which spatially organized context information becomes temporally organized as animals experience an environment* (Buzsaki, 2006; Buzsaki and Moser, 2013). Specifically, this type of temporal relationship may confer a high level of temporal alignment (and hence accuracy) between expected and experienced context information, and this in turn should increase the accuracy of their comparison.

Since multiple brain areas demonstrate rhythmic neural activity, neural oscillations are likely a fundamental mechanism for coordinating neural activity across the brain in the service of adaptive decisions, learning, and memory (e.g., Buzsaki, 2006; Fries, 2009; Monaco et al., 2011; Penner and Mizumori, 2012b). Numerous laboratories have now reported that synchronous neural activity (in particular coherence of the theta rhythm) can be detected within and between memory-related brain structures such as the hippocampus, striatum, or prefrontal cortex (Tabuchi et al., 2000; Engel et al., 2001; Fell et al., 2001; Varela et al., 2001; Siapas et al., 2005; DeCoteau et al., 2007a; Womelsdorf et al., 2007). For example, hippocampal theta activity can become synchronized with place cell firing, resulting in coordinated timing of spatial coding (O’Keefe and Recce, 1993; Gengler et al., 2005). Also, theta oscillations within the striatum can become entrained to the hippocampal theta rhythm (Berke et al., 2004; DeCoteau et al., 2007a). Stimulating the striatum can induce hippocampal theta activity (Sabatino et al., 1985) and increases high frequency theta power, which is thought to be important for sensorimotor integration (Hallworth and Bland, 2004). Also, hippocampal and striatal activity theta activity become increasingly coherent during goal-directed navigation (Allers et al., 2002; DeCoteau et al., 2007a). When neural activity is disrupted in the striatum via D2 receptor antagonism, striatal modulation of high frequency hippocampal theta activity is also disrupted. The result is that motor and spatial/contextual information is not integrated, and task performance is impaired (Gengler et al., 2005).

Particularly intriguing is a finding common to both hippocampus and striatum, and that is that synchronous activity occurs in specific task-relevant ways (e.g., Hyman et al., 2005; Jones and Wilson, 2005) particularly during times when rats are said to be engaged in decision making (e.g., Benchenane et al., 2010). For example, striatal theta is modified over the course of learning on an egocentric T-maze task, increasing in coherence as the rat chooses and initiates turn behavior (DeCoteau et al., 2007a,b). Rats that learn the task develop an antiphase relationship between hippocampal and striatal theta oscillations, while rats that do not learn the task also do not show this type of theta relationship. This coherence has also been observed during striatal-dependent classical conditioning (Kropf and Kuschinsky, 1993). Coherent theta oscillations across distant brain structures can be enhanced with application of dopamine (Benchenane et al., 2010). Dopamine, then, may play a crucial role in coordinating ensemble activity across brain areas during times of decision making during navigation. Functionally, this type of control by dopamine suggests that information about the saliency of reward may determine which brain systems become synchronized (and desynchronized). This in turn guides the nature and type of information that is used to update memories and to determine future responses.

Task demands, then, seem to dictate the nature of neural synchrony across distal brain structures, and this synchrony may take the form of comodulation of existing theta and gamma rhythms as well as the generation of an additional rhythm. The latter was illustrated in a recent study by Fujisawa and Buzsaki (2011). They demonstrated that the existence of very low frequency (4 Hz) entrainment of local field potentials, e.g., the 7–12 Hz theta oscillation, that emerges only during phases of a maze task when rats made decisions (i.e., in the stem of a T-Maze). During decision periods, the 4 Hz rhythm was phase locked to theta oscillations in both the prefrontal cortex and VTA. Some of the individual prefrontal and VTA neurons were also phase locked to hippocampal theta oscillation at this time. Importantly the 4 Hz rhythm was present only during a decision making period when theta oscillations were also present. The findings of this study suggest that a 4 Hz rhythm may coordinate activity in distal brains structures specifically as animals make decisions during goal-directed navigation.

Assuming that “orchestrating” rhythms such as the 4 Hz rhythm also incorporate hippocampal neural activity, place field remapping is expected to have effects that extend well beyond hippocampal computations. When an expected context changes, evidence shows that place fields change by either increasing or decreasing firing rates and/or changes in their spatial specificity and reliability. This period of place field change corresponds to the period of uncertainty that is generated with by the context change. Therefore, as uncertainty decreases, place fields become more stable, and this in turn should result in the generation of more stable comodulation of specific frequencies of EEG across brain structures.

To fully understand the role of the hippocampus in episodic memory requires an understanding of how hippocampus relates event-specific details to particular temporal features of a task. Recently many (Manns et al., 2007b; Pastalkova et al., 2008; Gill et al., 2011; McDonald et al., 2011; Kraus et al., 2013) have suggest the existence of “time cells” in addition to place cells in hippocampus. These studies demonstrate that the timing of cell firing by a subpopulation of hippocampal neurons is not directly related to spatial or behavioral metrics such as distance, location, or running speed but rather they appear tuned to the timing of task-relevant events. Another challenge for future research is to better define how hippocampal context prediction error signals are interpreted and valued by neural systems responsible for decisions based on the most recent context analysis, and ultimately, by neural systems that update memories (Lisman and Grace, 2005; Penner and Mizumori, 2012a,b). The midbrain dopaminergic system is likely involved in this assessment process since dopamine neurons are not only known to respond to changes in the learned value of rewards, and to cues that predict future rewards (Schultz et al., 1997), but also they respond to changes in reward values in a context-dependent manner when rats perform a hippocampal-dependent task (Puryear et al., 2010).

Dopamine neural responses to the expectation of rewards seem to be regulated at least in part by prefrontal cortex (Jo et al., 2013), suggesting that the prefrontal cortex may relay to dopamine neurons information based on stored memories about past consequences of behavior. Penner and Mizumori (2012b) recently reviewed an extensive literature that describes how dopamine cell responses to hippocampal-based context information can come to regulate subsequent choices and response selection as information is processed through iterative striatal-to-cortex information loops (Haber et al., 2000). The result of this sequence of striatal-cortical processing is the determination of the degree to which expected behavioral outcomes occurred, and the updating of long-term memory. The latter, in turn, updates the definition of expected context features that cortex subsequently provides hippocampus (Penner and Mizumori, 2012a,b; Mizumori and Jo, 2013). Indeed Martig and Mizumori (2011) have shown that inactivation of the VTA results in unstable hippocampal place fields and behavioral errors when rats perform a hippocampal-dependent spatial task. Since the outcome assessment by VTA impacts the subsequent stability of hippocampal neural codes and behavioral accuracy on a spatial working memory task, it is likely that hippocampus and the VTA-striatal circuitry comprise key components of an adaptive loop of neural processing that allows organisms to continuously update memories and memory representations according to the outcomes of choices made within circumscribed epochs of time.

## Homeostatic Regulation of Neural Signals of Prediction Errors

An emerging view is that the brain has evolved in large part to allow organisms to accurately predict the outcomes of events and behaviors (e.g., Llinas and Roy, 2009; Buzsaki, 2013; Buzsaki and Moser, 2013; Mizumori and Jo, 2013). More specifically, it has been suggested that organisms have been able to adapt to environments and societies of increasing complexity because brains evolved more complex neural circuitry that support the ability to make dynamic and conditional decisions and predictions. These neural ensembles evolved to retain information over times of varying scales depending on the desired goal. Different brain areas are known to generate and retain sequences of information, and this ability can be accounted for by state-dependent changes in network dynamics (Mauk and Buonomano, 2004), internally generated oscillatory activity (Pastalkova et al., 2008), and/or dedicated “time cells” (Kraus et al., 2013). Thus, many elemental features of prediction analyses seem to be intrinsic, or self-generated. This property is likely very important for it may provide a mechanism by which prediction analyses can occur automatically so that organisms naturally seek control of the outcomes to their behaviors. What, then, might be the mechanism of a self-generated, and thus auto-regulated, brain prediction system? It is suggested here that such a mechanism may to some extent mirror principles of self-regulation at synaptic and neural circuit levels (e.g., Turrigiano, 1999, 2008, 2011; Marder and Prinz, 2003; Turrigiano and Nelson, 2004; Marder and Goaillard, 2006; Shetty et al., 2012; Mizumori and Jo, 2013).

Marder and Goaillard (2006) suggested that homeostatic neuroplasticity may be nested: calcium sensors may monitor neural firing rates, then up or down regulate the availability of glutamate receptors to ramp up or down firing rates toward an optimal firing rate set point. Groups of neurons or neural networks may sense changes in firing collectively to regulate experience-dependently population activity levels and patterns of activation. In this way homeostatic plasticity enables groups of neural circuits to find a balance between flexible and stable processing as needed to learn from experiences, and to be responsive to future changed inputs. The details of how networks of cells or their connections engage in homeostatic regulation remain to be discovered. Nevertheless, it is worth noting that homeostatic regulation at the neural systems level is clearly evident from studies of brain development, as well as from studies of reactive or compensatory neuroplasticity mechanisms that occur in response to experience (e.g., sensorimotor learning; Froemke et al., 2007) or brain injury (e.g., brain trauma or addiction; Robinson and Kolb, 2004; Nudo, 2011). While specific homeostatic neural plasticity mechanisms have not been used to account for complex learning, current theories of reinforcement- and context-based learning and memory commonly rely on the autoregulation of feedback loops.

A homeostatic framework could apply to the autoregulation of prediction analyses, which in turn will impact regulation of future decisions and memories. Such a framework includes *variables* that are monitored by *sensors* and then regulated by *controllers*, and thus it likely involves multiple, interactive, and hierarchically organized (auto-regulated) information loops analogous to what was described by Buzsaki (2013). At the cellular or synaptic levels, homeostatic plasticity mechanisms (Turrigiano et al., 1998; Marder and Goaillard, 2006; Turrigiano, 2011) may regulate cell excitability around a neural activity *set point* such that neurons retain maximal responsivity to future inputs. This process enables neurons to achieve a balance between synaptic stability and flexibility. Changes in calcium flux appear to be an important part of the sensing system that determines the current level of firing. It is hypothesized here that a prediction error, or mismatch signal, may result in higher or lower firing rates, at which time *controller* mechanisms should be engaged to bring the firing rates back to set point levels. Indeed reward prediction errors are illustrated by transient and significant reduced or elevated neural firing depending on the valence of the error (Schultz et al., 1997). Future research should focus on understanding the enabling and restorative mechanisms of prediction error signals. Of particular interest are mechanisms by which cortical memory may impact the threshold for signaling prediction errors. One cortical area of interest is the prefrontal cortex given (a) its intrinsic recurrent circuitry and detailed excitatory and inhibitory extrinsic connections (as reviewed in Arnsten et al., 2012) with both hippocampal/temporal lobe and reward valuation systems, and (b) given its role in attention and working memory (e.g., Fuster, 2006, 2008, 2009). That is, prefrontal cortex may orchestrate and coordinate the level of neural excitability in different prediction error brain areas according to homeostatic principles and in this way, bias the nature of the outputs of connected brain areas according to experience and recent outcomes of decisions. Prefrontal cortex also has strong functional connections with other cortical memory areas (e.g., parietal and temporal cortex), and is thus strategically positioned to influence long-term memory updating based on prediction error analyses. In this way the most recent memories can be fed forward to hippocampus for future context evaluation.

Although it is reasonable to assume that the prefrontal cortex controls or biases neural signaling in distal prediction brain regions, it should be noted that other sources of control of cell excitability may arise via direct interconnections amongst the multiple prediction detection areas of the brain. For example, a prediction error signal from the hippocampus could be transmitted to midbrain-striatal neurons along pathways that do not necessarily include the prefrontal cortex. Indirect support for this idea come from observations that conditions that produce error messages in the hippocampus change reward responses of dopamine neurons (Puryear et al., 2010; Jo et al., 2013), phasic theta comodulation is observed between hippocampus and striatum (DeCoteau et al., 2007a) during decision tasks, and comodulation of neural activity has been reported between prefrontal cortex and parietal cortex (Diwadkar et al., 2000). However, the identification of such correlations does not necessarily mean that this is the source of the comodulation or neural synchrony. Finding the source of neural regulation remains a challenge for the general field of systems neuroscience, one that may soon have answers with continued development of new methodologies such as optogenetic analyses.

In sum, homeostatic regulatory processes may contribute to the automatic and continuous self-regulatory nature of prediction error analysis, and ultimately decision making and episodic memory. Such a naturally adaptive mechanism optimizes the contribution of different types of prediction error signals to future decisions and actions according to the pattern of recent successes and failures in prediction.

## Conflict of Interest Statement

The author declares that the research was conducted in the absence of any commercial or financial relationships that could be construed as a potential conflict of interest.
